# Fighting Malaria

**Published:** 1917-05

**Authors:** 


### Fighting Malaria.

A systematic effort is being made by the government to stamp out malaria in Peru, and if persistently followed will likely prove effective. The work is classified under four heads: Treatment for patients, protection of people living in places where malaria is recognized as epidemic, destruction of germ-transmitting insects, and drainage and other special treatment of swampy lands.

In this country the necessity for preventing malaria is being recognized, and in limited areas something is being done in the way of systematic effort, but Peru seems to be far ahead of this country in its determination to combat the spread of malaria.

The article in the TEXAS MEDICAL JOURNAL last month from Dr. A. Caswell Ellis, of the University of Texas, shows the enormous waste in East Texas and how it can be effectually stopped by the right sort of concert of action.

It is little short of criminal for any country to permit health-destroying conditions to exist when they can be prevented. Besides, from a purely business standpoint, no country can afford it. Some of the richest portions of Texas, Louisiana, Arkansas, and other Southern States have deteriorated to almost worthlessness due to unchecked malaria.

This country was greatly interested in the health conditions of Cuba and shocked at what it discovered there,—so shocked that it went to work and soon worked marvelous improvements—but is apparently oblivious to or unconcerned about just as bad conditions found in parts of this country.

